# Eculizumab as a Disease‐Modifying Therapy in Chronic Inflammatory Demyelinating Polyneuropathy (CIDP): A Case Report

**DOI:** 10.1111/jns.70010

**Published:** 2025-03-02

**Authors:** Edoardo Dalmato Schilke, Diletta Cereda, Maria Letizia Fusco, Lorenzo Stanzani, Laura Marzorati, Michela Ripolone, Letizia Bertolasi, Maura Frigo, Franco Molteni, Nico Farina, Carlo Ferrarese, Guido Cavaletti, Claudia Balducci

**Affiliations:** ^1^ PhD Program in Neuroscience, School of Medicine and Surgery University of Milano‐Bicocca Milan Italy; ^2^ Neurology Clinic, Foundation IRCCS San Gerardo Dei Tintori Monza Italy; ^3^ Neuromuscular and Rare Diseases Unit, Department of Neuroscience Fondazione IRCCS Ca' Granda Ospedale Maggiore Policlinico Milan Italy; ^4^ Department of Rehabilitation Medicine Valduce Hospital ‐ Villa‐Beretta Costa Masnaga Italy; ^5^ School of Medicine and Surgery and Milan Centre for Neuroscience (NeuroMI), University of Milano‐Bicocca Milan Italy; ^6^ Experimental Neurology Unit School of Medicine and Surgery, University of Milano‐Bicocca Milan Italy

**Keywords:** chronic inflammatory demyelinating polyneuropathy (CIDP), complement inactivating agents in CIDP, complement system in CIDP, eculizumab in CIDP, refractory CIDP

## Abstract

**Background and Aims:**

Chronic inflammatory demyelinating polyneuropathy (CIDP) is a rare immune‐mediated disorder; about 20%–30% of patients do not adequately respond to first‐line treatments (immunoglobulins, therapeutic plasmapheresis, and corticosteroids) posing diagnostic and therapeutic challenges.

**Case Report:**

We report the case of a 58‐year‐old man diagnosed with CIDP. During follow‐up, he progressively became refractory to all first‐line treatments. Therefore, 20 months after the diagnosis, an add‐on therapy with Rituximab was tried. Despite previous works supporting the use of Rituximab in refractory CIDP, in our case, the patient experienced relapses and progressive increases in disability. After the exclusion of potential CIDP mimics and considering the histological findings that showed complement activation, we opted for an innovative therapeutic approach with Eculizumab that granted a significant clinical and neurophysiological benefit that persists after 7 months of follow‐up.

**Interpretation:**

CIDP pathogenesis is characterized by a complex interplay of different immunopathological mechanisms, and the complement system may play a major role. The present case supports the role of complement‐dependent toxicity in CIDP and suggests that complement‐targeted therapies may represent a well‐tolerated and effective alternative for CIDP treatment.

## 1 Background and Aims

Chronic inflammatory demyelinating polyneuropathy (CIDP) is a rare immune‐mediated disorder causing demyelination and axonal damage of peripheral nerves. It is characterized by symmetrical limb weakness, loss of sensation, dysesthesia, and loss of tendon reflexes that may have either a relapsing and remitting or progressive clinical course longer than 8 weeks. The first‐line therapies for CIDP are intravenous immunoglobulins (IVIg), therapeutic plasmapheresis (TPE), and corticosteroids. However, approximately 20%–30% of patients with CIDP do not adequately respond to these therapies. The main reason for the presumed failure of standard therapy is an incorrect diagnosis. Therefore, a diagnosis of refractory CIDP requires the exclusion of several mimics [1]. In refractory CIDP, there are no randomized clinical trials to guide clinicians in the selection of the optimal treatment once first‐line therapies fail. Several case reports/series suggest that patients benefit from rituximab and/or cyclophosphamide among other chemotherapeutic agents. However, approximately 15% of patients remain refractory to all treatment options. Risk factors for patients being refractory are not completely clear, although irreversible axonal degeneration has been considered a possible factor contributing to refractoriness [2]. Patients with refractory CIDP experience continued worsening of their disease either with relapses or with a progressive course, despite treatment efforts. The lack of response to treatment has a significant physical and psychosocial impact on patients, highlighting the need for effective therapies to address the unmet needs of those refractory CIDP patients.

## 2 Case Report

A 58‐year‐old Caucasic male was hospitalized for a subacute (about 2 months) onset of distal numbness and weakness in his four limbs (INCAT scale at admission: 4). Electromyography (EMG) showed a symmetric sensorimotor demyelinating polyradiculoneuropathy. CSF was acellular, with a protein content equal to 55 mg/dL. Laboratory analyses showed a serum IgG‐kappa monoclonal gammopathy and an HBV serology compatible with inactive infection. On suspicion of acute inflammatory demyelinating polyradiculoneuropathy (AIDP), he underwent IVIg treatment at 0.4 g/Kg for 5 days with clinical improvement (INCAT scale: 2).

Two months later, the patient was again hospitalized due to a worsening of upper and lower limb weakness and diffuse paraesthesia (INCAT scale: 3). EMG showed a worsening of nerve conduction parameters consistent with a symmetric sensorimotor demyelinating polyneuropathy (Table 1‐timepoint a). Ancillary tests ruled out other causes of peripheral neuropathy, particularly serum antibodies anti‐MAG and anti‐ganglioside (GM1, asialo‐GM1, GM2, GD1a, GD1b, and GQ1b) and total body angio‐CT scan resulted negative. Clinical and electrodiagnostic findings were consistent with a diagnosis of acute‐chronic inflammatory demyelinating polyneuropathy (A‐CIDP) according to EFNS/PNS criteria. The patient underwent a IVIg treatment of 0.4 g/kg for 5 days with benefit (INCAT scale: 2). After discharge, although the patient remained stable, monthly IVIg and a chronic corticosteroid treatment were required owing to end‐dose worsening of neuropathic symptoms.

**TABLE 1:** Reported electrophysiological studies of motor and sensory nerves performed at different time points (a, b, c, d, e, f) during follow‐up.

		Timepoint a	Timepoint b	Timepoint c	Timepoint d	Timepoint e	Timepoint f
Motor study distal latency (ms)							
Median nerve	R				10.6	13.5	9.3
	L				7.9	10.4	8.7
Ulnar nerve	R	9.2	6.1	7.7	9.5	10.7	6.9
	L		6.7		8.4	9.9	8.3
Peroneal nerve	R	7.1	6.7	6.2	7.5^a^	9.1^a^	9.4^a^
	L	7.1	10.5	9.1	ND	10.9^a^	11.4^a^
Tibial nerve	R	12.8	28.2	23.4	ND	ND	ND
	L		14.4		ND	ND	ND
Proximal latency (ms)							
Median nerve	R				27.5	27.8	20.7
	L				25.9	28.8	20.7
Ulnar nerve	R	16.1	13.9	20.9	29.7	32.8	22.0
	L		14.4		26.5	32.2	24.0
Peroneal nerve	R	15.8	22.0	31.9	15.9^a^	20.8^a^	23.8^a^
	L	16.9	23.8	25.9	ND	19.2^a^	15.1^a^
Tibial nerve	R	24.8	60.3	63.7	20.2	ND	ND
	L		43.0		21.4	ND	ND
Conduction velocity (m/s)							
Median nerve	R				13.8	17.5	19.3
	L				13.7	14.1	19.3
Ulnar nerve	R	35.2	26.7	17.5	9.9	10.2	15.9
	L		31.1		12.7	11.2	14.6
Peroneal nerve	R	40.8	22.1	17.1	12.5^a^	7.7^a^	6.3^a^
	L	38.2	25.5	25.1		10.9^a^	13.4^a^
Tibial nerve	R	38.5	13.7	10.2			
	L		15.4				
Distal amplitude (mV)							
Median nerve	R				3.0	5.7	6.9
	L				3.3	5.4	8.2
Ulnar nerve	R	4.7	9.4	6.1	6.6	3.1	7.6
	L		10.1		7.5	4.0	7.1
Peroneal nerve	R	2.3	3.4	1.6	1.8^a^	0.2^a^	1.1^a^
	L	3.6	3.7	0.7		0.4^a^	1.3^a^
Tibial nerve	R	2.4	1.6	0.5	ND		
	L		1.9		ND		
Proximal amplitude (mV)							
Median nerve	R				0.6	1.5	4.8
	L				0.6	0.2	3.5
Ulnar nerve	R	4.7	6.4	2.4	0.5	0.6	4.0
	L		4.3		0.7	0.5	6.2
Peroneal nerve	R	2.0	0.8	0.1	1.7^a^	0.2^a^	0.2^a^
	L	3.5	1.9	0.2		0.2^a^	0.4^a^
Tibial nerve	R	1.9	0.1	0.3	0.1		
	L		0.1		0.2		
F‐wave (ms)							
Median nerve	R						
	L						
Ulnar nerve	R	44.8	73.8	73.9			
	L						
Peroneal nerve	R						
	L	66.3					
Tibial nerve	R						
	L						
Sensory study distal latency (ms)							
Median nerve	R				ND	ND	ND
	L				ND	ND	ND
Ulnar nerve	R	2.8	ND	ND	ND	ND	ND
	L		ND	ND	ND	ND	ND
Sural nerve	R	2.4	ND	ND	ND	ND	ND
	L	2.7	ND	ND	ND	ND	ND
Superficial peroneal nerve	R				ND		
	L				ND		
Conduction velocity (m/s)							
Median nerve	R						
	L						
Ulnar nerve	R	53.3					
	L						
Sural nerve	R	54.3					
	L	55.4					
Superficial peroneal nerve	R						
	L						
Distal amplitude (mV)							
Median nerve	R				ND	ND	ND
	L				ND	ND	ND
Ulnar nerve	R	2.1	ND	ND	ND	ND	ND
	L		ND	ND	ND	ND	ND
Sural nerve	R	0.2	ND	ND	ND	ND	ND
	L	1.4	ND	ND	ND	ND	ND
Superficial peroneal nerve	R				ND		
	L				ND		

Abbreviations: “L”: left, “ND”: nondetectable, “.”: not evaluated, “R”: right.

^a^ Derived from anterior tibial muscle instead of extensor digitorum brevis.

**FIGURE 1:**
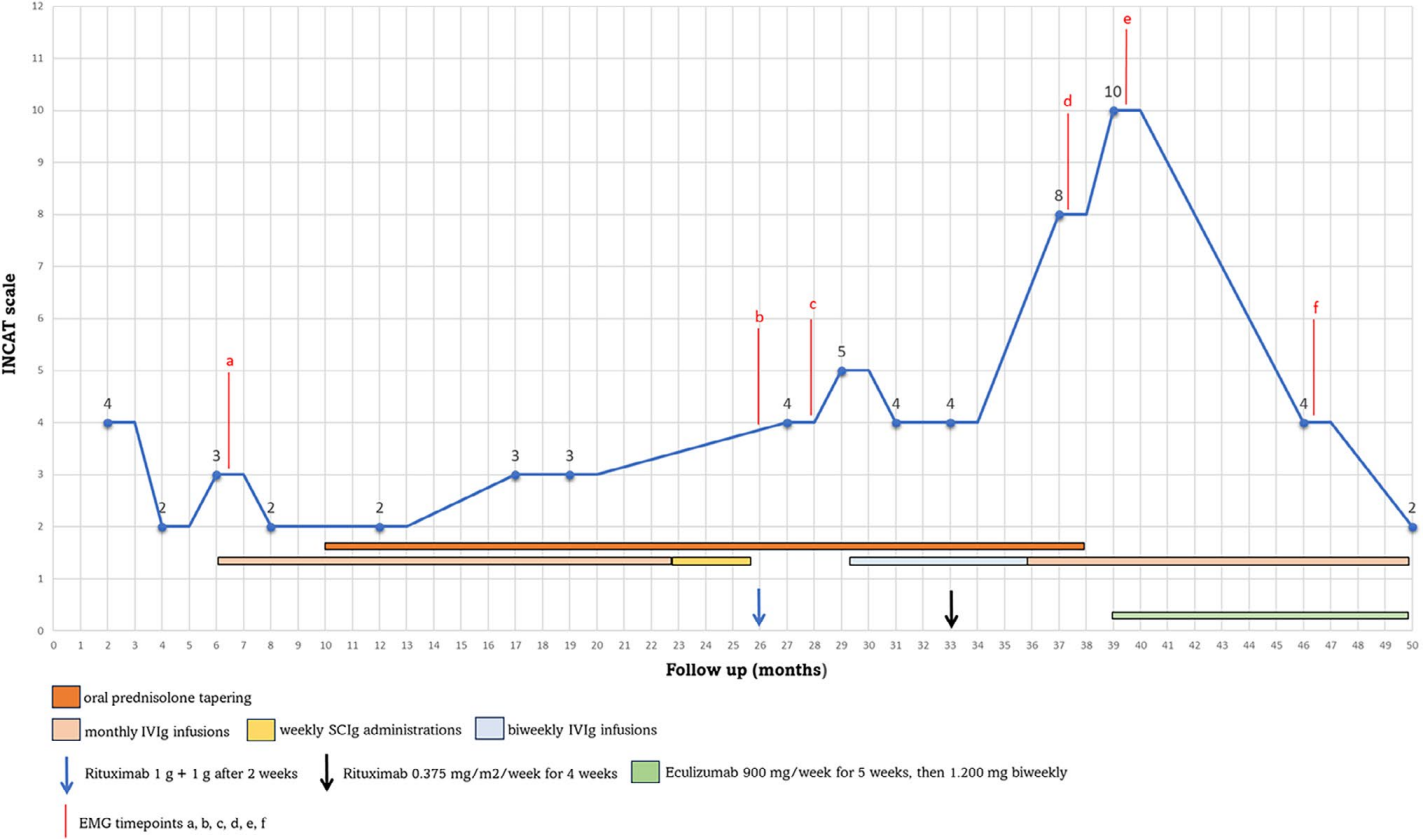
The course of CIDP (evaluated with total ONLS) in our patient with treatments. Time points of electromyography (EMGs) are also reported through the same codes (timepoint a, b, c, d, e, f) used in the article. IVIg, intravenous immunoglobulins; SCIg, subcutaneous immunoglobulins.

**FIGURE 2:**
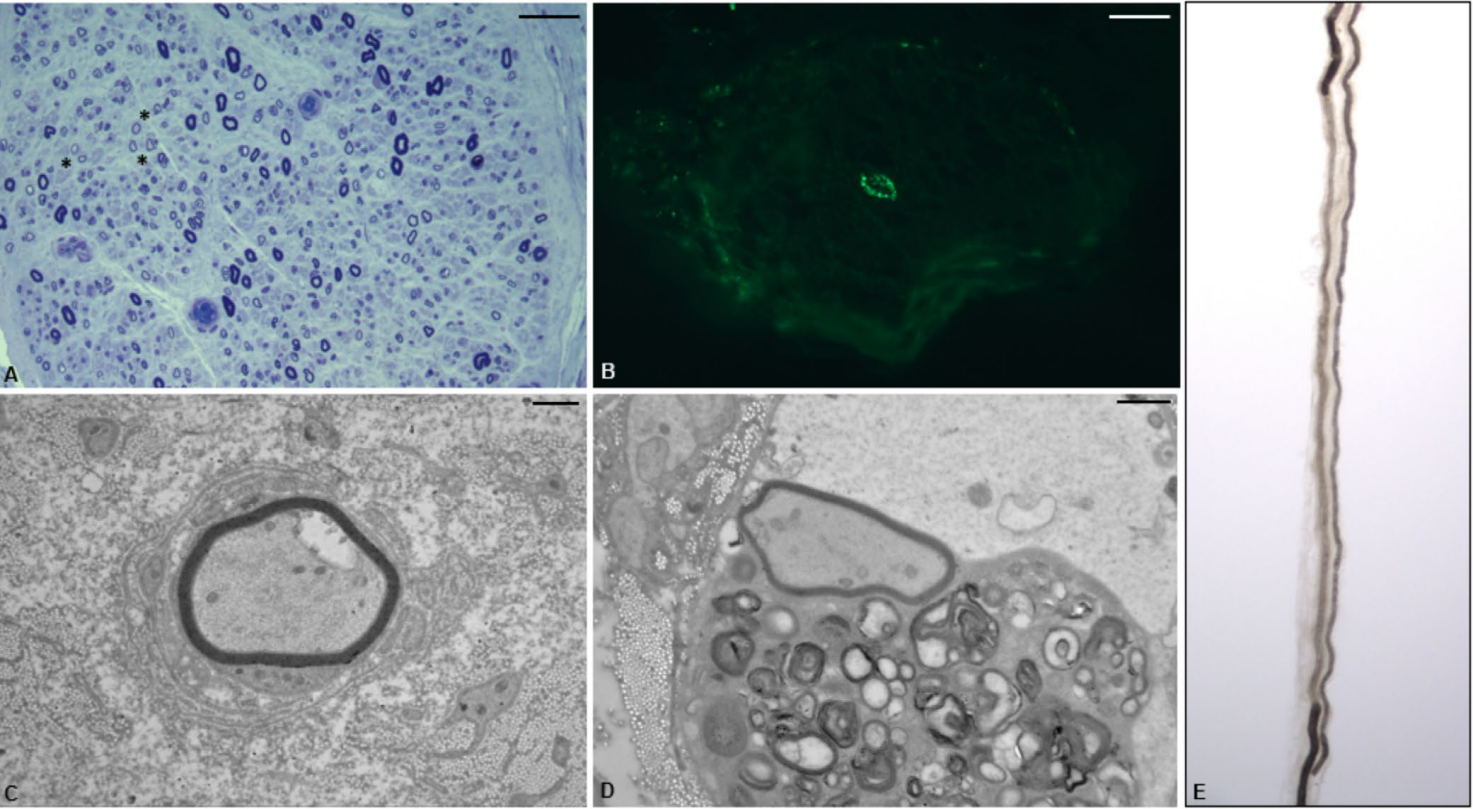
Sural nerve biopsy analyses, light and ultrastructural examination. Moderate reduction in myelinated fibers, with numerous fibers displaying thin myelin sheaths, and asterisks (A). Endoneurial C5b‐9 positive vessel (B). Small onion bulb (C). Macrophage adhering to axon (D). Demyelination in a single‐fiber analysis (E). Bar: (A, B) 25 μm. (C, D) 1 μm.

Ten months later, despite the same treatment scheme, the patient started to experience a progressive and relapsing worsening of his disease. As shown in Figure 1, several treatment attempts resulted ineffective. At first, subcutaneous immunoglobulins (SCIg) were prescribed (from 0.2/g/kg/week up to 0.33 g/kg/week) without benefits. Then, an add‐on therapy with Rituximab was tried, associating entecavir 0.5 mg/day to prevent HBV reactivation. Despite one first cycle of Rituximab 1 g + 1 g after 2 weeks and a second cycle (7 months later) of Rituximab 0.375 mg/m^2^/week for 4 weeks, the patient experienced a progressive worsening of lower limbs weakness that prevented deambulation (INCAT scale: 8). During this period, subsequent EMGs evidenced a progressive worsening of nerve conduction parameters (Table 1—timepoint b, c, d). About 20 months after the diagnosis, the patient was hospitalized for new diagnostic assessments. Bone marrow biopsy was negative for plasma cell expansion, body imaging (chest, abdomen and pelvis PET/angio‐CT scans), brain and spinal cord MRI, the spit Transthyretin‐Related Hereditary Amyloidosis Test, serum onconeural antibodies F resulted negative, and serum vascular endothelial growth factor (VEGF) resulted normal. The light and ultrastructural analyses of the sural nerve biopsy revealed significant findings in favor of CIDP; notably, complement deposition with a granular pattern was observed in the walls of endoneurial blood vessels (Figure 2).

During hospitalization, clinical disability kept worsening to the point that the patient was bedridden; neurological examination revealed severe tetraparesis, generalized areflexia, severe distal paresthesias, and numbness in the lower and upper limbs; cranial nerve examination was negative (INCAT scale: 10). A new EMG showed a severe symmetric axonal involvement determining a pronounced reduction of distal muscle action potentials (Table 1—timepoint e). After having obtained informed consent and vaccination for Haemophilus Influentia B, Pneumococcus (13–20) and *Neisseria Meningitidis* (A‐C‐W‐Y‐B), Eculizumab was infused, 900 mg every week for 5 weeks followed by biweekly 1.200 mg. During the first 10 weeks of treatment, the patient underwent antibiotic prophylaxis with amoxicillin 2 g/day. One month after Eculizumab initiation, the patient presented a dramatic improvement in his strength in the four limbs (without any further relapse) and had an almost complete disappearance of limb numbness and dysesthesias. Seven months later, he was fully ambulatory, requiring mild assistance for daily activities (INCAT scale: 4). EMG showed a congruent improvement in nerve conduction parameters (Table 1—timepoint f). In the following months, the patient underwent rehabilitative treatments with wearable robotics for gait training and muscle electrostimulation. At present, neurological examination still reveals mild distal weakness in the upper limbs (in the absence of functional limitations) and in the lower limbs (he can walk with bilateral steppage outdoors without aid). Tendon reflexes are still diffusely absent. He complains of mild distal paresthesias and numbness in the upper and lower limbs (INCAT scale: 2). The patient is prosecuting rehabilitative treatment as an outpatient and has stopped using a leg‐foot orthosis.

## 3 Interpretation

A substantial proportion of patients with CIDP are refractory to first‐line treatments. New drugs tried in CIDP are first aimed at refractory patients. These therapeutic approaches still lack head‐to‐head comparisons, and, since CIDP is a heterogenous entity and treatment responses vary widely from one patient to another, it is conceivable that a large therapeutic armamentarium will be needed to treat refractory patients.

CIDP pathogenesis is characterized by a complex interplay of different immunopathological mechanisms, involving cellular, humoral, and complement pathways, which culminate in a highly variable pattern of peripheral nerve damage. However, several studies suggested that the complement system plays a major role [3]. Preclinical studies showed that the passive transfer of IgG with complement C3 reactivity from CIDP patients into rats results in nerve conduction impairment and demyelination; also, the administration of soluble complement receptor one (sCR1), which is a natural complement inhibitor, suppresses the disease. Before us, some clinical studies highlighted in vivo the role of complement‐dependent cytotoxicity in CIDP pathogenesis. By evaluating nerve biopsy specimens from CIDP patients, one study showed the deposition of complement‐fixing immunoglobulins M (IgM) in seven out of seven samples, and the deposition of C3 in six samples; a subsequent study evidenced the presence of C3d, which is part of the immune complex, on the myelin sheaths in two out of four samples. In a recent case report of a CIDP patient with anti‐LM1 antibodies, an immunohistochemical analysis revealed the deposition of C9 neoepitope, a component of MAC, on the majority of the length of the nerve compact myelin sheath. Finally, one study evidenced higher serum and CSF levels of C5a and terminal complement complex (TCC) in a small cohort of newly diagnosed CIDP patients compared with controls. In addition, C5a and TCC levels correlated with disease activity.

Eculizumab is a monoclonal antibody that binds to complement factor C5 and prevents its cleavage to C5a and C5b, thereby inhibiting membrane attack complex (MAC) formation. So far, Eculizumab has given disappointing results in phase II/III clinical trials where it was used as an add‐on therapy in acute inflammatory demyelinating polyradiculoneuropathy (AIDP) [4]. However, no clinical trial has yet investigated Eculizumab's role in CIDP, whereas preliminary results from a phase II open‐label study [5] demonstrated a favorable benefit/risk profile in refractory patients of an add‐on therapy with a C1s inhibitor, which is part of the activation of the classical complement pathway (Riliprubart). Therefore, one phase 3 clinical trial (NCT06290128) is currently ongoing to confirm Riliprubart's add‐on therapy efficacy in CIDP patients refractory to first‐line treatments, and another phase 3 clinical trial (NCT06290141) is investigating the safety and efficacy of a Riliprubart monotherapy versus an active comparator (IVIg).

The successful use of Eculizumab in refractory CIDP, as presented here, supports the role of complement toxicity in CIDP and suggests that complement‐targeted therapies may represent a well‐tolerated and effective alternative for CIDP treatment. However, this should be confirmed by future clinical trials conducted in larger cohorts of patients with relapsed and refractory CIDP. Further studies will also be needed to define the characteristics of patients with refractory CIDP who are more willing to respond to anti‐complement agents rather than other innovative drugs with different targets, such as B‐cell‐depleting therapies.

## Data Availability

Data sharing not applicable to this article as no datasets were generated or analysed during the current study.
